# Effects of essential oil extracted from *Artemisia argyi* leaf on lipid metabolism and gut microbiota in high-fat diet-fed mice

**DOI:** 10.3389/fnut.2022.1024722

**Published:** 2022-11-03

**Authors:** Kaijun Wang, Jie Ma, Yunxia Li, Qi Han, Zhangzheng Yin, Miao Zhou, Minyi Luo, Jiayi Chen, Siting Xia

**Affiliations:** ^1^College of Animal Science and Technology, State Key Laboratory for Conservation and Utilization of Subtropical Agro-bioresources, Guangxi University, Nanning, Guangxi, China; ^2^Animal Nutritional Genome and Germplasm Innovation Research Center, College of Animal Science and Technology, Hunan Agricultural University, Changsha, Hunan, China; ^3^Agricultural Service Center, Xiaolan Town, Zhongshan, Guangdong, China; ^4^Academician Workstation, Changsha Medical University, Changsha, Hunan, China

**Keywords:** *Artemisia argyi*, high fat, lipid, gut, microbiota

## Abstract

Artemisia argyi leaf is a well-known species in traditional Chinese medicine, and its essential oil (AAEO) has been identified to exert various physiological activities. The aim of this study was to investigate the effects of AAEO on lipid metabolism and the potential microbial role in high-fat diet (HFD)-fed mice. A total of 50 male mice were assigned to five groups for feeding with a control diet (Con), a high-fat diet (HFD), and the HFD plus the low (LEO), medium (MEO), and high (HEO) doses of AAEO. The results demonstrated that dietary HFD markedly increased the body weight gain compared with the control mice (*p* < 0.05), while mice in the HEO group showed a lower body weight compared to the HFD group (*p* < 0.05). The weight of fatty tissues and serum lipid indexes (TBA, HDL, and LDL levels) were increased in response to dietary HFD, while there was no significant difference in AAEO-treated mice (*p* < 0.05). The jejunal villus height was dramatically decreased in HFD-fed mice compared with the control mice, while HEO resulted in a dramatically higher villus height than that in the HFD group (*p* < 0.05). Microbial α-diversity was not changed in this study, but β-diversity indicated that microbial compositions differed in control, HFD, and EO subjects. At the genus level, the relative abundance of *Bacteroides* was greater (*p* < 0.05) in the feces of the Con group when compared to the HFD and EO groups. On the contrary, the abundance of *Muribaculum* was lower in the Con group compared to the HFD and EO groups (*p* < 0.05). Although the *Muribaculum* in the EO group was lower than that in the HFD group, there was no statistically notable difference between the HFD and EO groups (*p* > 0.05). Simultaneously, the relative abundance of *Alistipes* (*p* < 0.05) and *Rikenella* (*p* < 0.05) was also dramatically higher in the Con group than in the HFD and EO groups. The abundance of *norank_f__norank_o__Clostridia_UCG-014* was lower in the HFD or EO group than in the Con group (*p* < 0.05). In conclusion, the results suggested that HEO could affect body weight and lipid metabolism without gut microbes in ICR mice, and it was beneficial for the structure of the jejunal epithelial tissue.

## Introduction

Obesity is a nutritional disorder caused by an imbalance between energy intake and expenditure (1). As economic development increased in developing countries, obesity and metabolic syndrome became more prevalent, primarily due to accelerated urbanization and nutritional changes; declining physical activity and genetics also played a role (2, 3). The prevalence of childhood obesity has increased by approximately 5% per decade over the past 50 years, not only among adults but also children (4). As fat accumulated in the body, dyslipidemia and metabolic syndrome would develop. An elevated level of lipids was associated with an increased risk of atherosclerosis and cardiovascular diseases (5). Atherosclerosis was closely associated with lipids and lipid-containing substances in the blood, of which cholesterol played the most important role (6). In humans, losing weight was a major way to reduce the risk of diabetes (7). It has been demonstrated that some anti-obesity drugs, such as orlistat and lorcaserin, as well as sustained-release tablets of phentermine/topiramate and naltrexone/bupropion are effective at reducing weight (8, 9). However, anti-obesity drugs may also cause gastrointestinal problems, weakness, mental disorders, and cardiovascular diseases, among others (10). Therefore, many experts predicted that natural products will provide hypolipidemic active factors that will intervene in obesity and its complications, as well as direct drug treatment.

There was a need for effective weight loss strategies and effective methods to prevent weight gain due to the current global obesity epidemic. A variety of gut microbial metabolites were necessary for gut microbiota to regulate host lipid metabolism, including short-chain fatty acids, secondary bile acids, and trimethylamine (11). Animal health depended on gut microbiota which were involved in digestion, metabolism, immunity, and defense against pathogens (12, 13). A better understanding of the interactions between host and gut microbes was crucial to study the complex relationship between host and microbiota. With the development of gene sequencing technology, we can explore the impact of changes in animal diet on the structure and function of intestinal microorganisms (14, 15). The molecules involved in this interaction could be measured, especially the microbiota-produced metabolites that were available to the host.

Looking for effective traditional Chinese medicine extracts is critical for obesity prevention, and it is still unclear how they impact the gut microenvironment. The leaves of *Artemisia argyi* were part of the Compositae family and were herbaceous perennial plants (16). It is well known that *A. argyi* leaves were the original source of Moxa floss (made from ground *A. argyi* leaves) in Moxibustion. The technique was widely known for its ability to diminish inflammation, relieve pain, promote blood circulation, and remove obstructions in channels through acupuncture and moxibustion therapy (17). *A. argyi* was beneficial for improving egg quality and increasing polyunsaturated fatty acids in egg yolk (18). Additionally, *A. argyi* leaf was used for preparing medical products, such as capsules and aerosols containing Moxa essential oil (16). Several studies had shown that *A. argyi* leaves were the main source of essential oil; it has antihistamine, phlegm-eliminating properties, cough-relieving properties, antifungal, and antiviral properties (19–21). Additionally, an analysis of the chemical composition, extraction yield, and quality evaluation of the essential oils extracted from *A. argyi* leaves (AAEO) has been conducted (22–24). Although AAEO was a geo-authentic medicine, there was relatively little research on it. The objective of this study was to investigate the effect of AAEO on lipid metabolism, body fat distribution, and gut microbes in a diet-induced obesity mouse model. Moreover, we also examined whether AAEO levels at different concentrations improved lipid metabolism. A systematic study was conducted using ICR mice to determine the effect of AAEO levels on fat metabolism and whether the effects were harmful or beneficial to the structure of the gastrointestinal tract.

## Materials and methods

### Animals and treatments

Animal experiments were conducted in accordance with the Hunan Agricultural University Institutional Animal Care and Use Committee (202105). Six-week-old male ICR mice were purchased from SLAC Laboratory Animal Central (Changsha, China). The mice were housed in a controlled environment after a 1-week adaptation period. AAEO was purchased from Jiangxi Hairui Natural Plant Co., Ltd., Jian, China.

A total of 50 male mice (29.40 ± 1.23 g) were divided into 5 groups at random, with 10 repetitions in each group. Mice were fed the control diet (Con), the high-fat diet (HFD), and the HFD-fed mice with the low (LEO), medium (MEO), and high (HEO) doses of AAEO. AAEO was dissolved in 4% Tween 80. In the LEO, MEO, and HEO groups, mice received 0.20, 0.40, and 0.80 ml/kg AAEO, respectively. The Con and HFD groups were given the same amount of physiological saline by oral gavage.

Fecal samples were obtained and kept at −80°C for examination. The mice were sacrificed under anesthesia. Finally, blood, subcutaneous adipose tissue (SAT), abdominal adipose tissue (AAT), epididymal adipose tissue (EAT), perirenal adipose tissue (PEAT), brown adipose tissue (BAT), liver, and jejunal tissue were collected for further examination.

### Analyzing blood samples for biochemical parameters

Serum extracted from blood samples using 3,000 rpm for 10 min at 4°C. Biochemical parameters of serum were tested using an automatic biochemistry analyzer (25), the index included total bile acid (TBA), glucose (Glu), total cholesterol (TC), triglycerides (TG), low-density lipoprotein (LDL), and high-density lipoprotein (HDL).

### Histological analysis

The jejunal tissue was removed and fixed in a 4% paraformaldehyde solution, then the fixed tissue was paraffin-embedded, and the jejunal tissue block was cut approximately 4-μm thick using a microtome and stained with hematoxylin and eosin (H&E) (26). The villus height was the distance from the villus tip to the crypt mouth, and crypt depth was the distance from the crypt mouth to the base of the crypt (27, 28).

### DNA extraction and microbiota analysis

As previously reported, DNA extraction and 16S ribosomal RNA amplification were carried out (29). Fecal samples were extracted for DNA with an E.Z.N.A. ^®^ soil DNA kit (Omega Biotek, Norcross, GA, USA) on the basis of the standard protocol. Using universal primers targeting the V3-V4 region 338F/806R, 16S rRNA from bacteria was amplified and sampled for sequencing using an Illumina Miseq PE300 platform (Illumina, SD, USA) (30). Sequence reads from the original sequence were uploaded to NCBI’s Sequence Read Archive. The 16S rRNA amplicon sequences have been deposited in the National Center for Biotechnology Information (NCBI) Sequence Read Archive (SRA)^1^ under accession number PRJNA861869.

### Statistical analyses

Statistical analyses between the means of each group were analyzed using one-way ANOVA (one-way analysis of variance) followed by Duncan comparison range tests through SPSS 22.0. The statistical significance level was set at *p* < 0.05.

## Results

### Body weight and organ index

**FIGURE 1:**
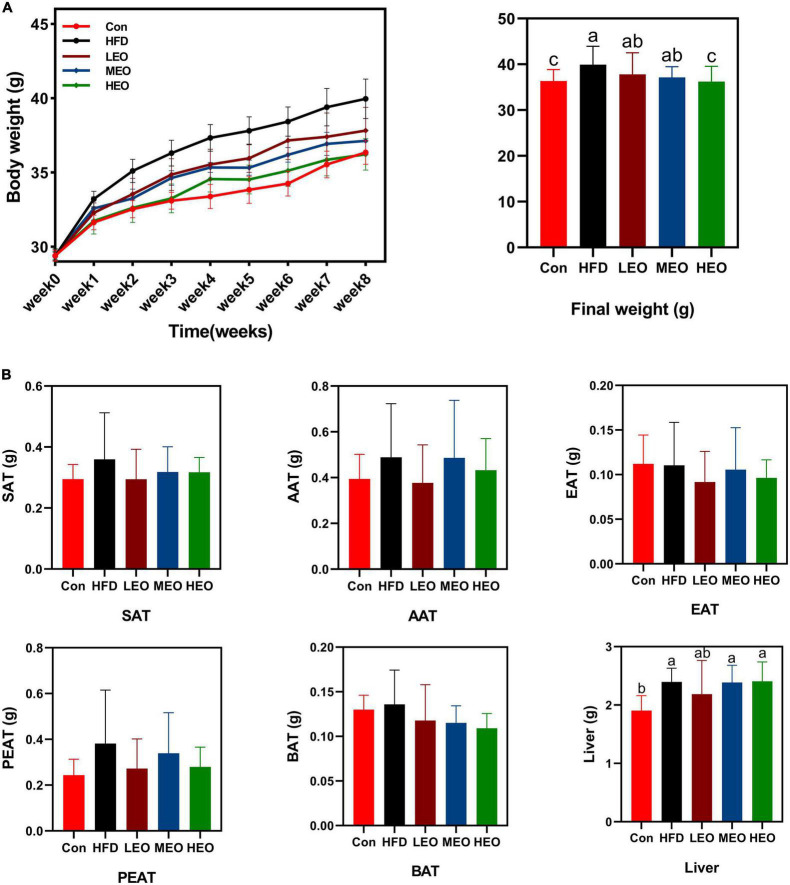
Effects of *Artemisia argyi leaves* (AAEO) on **(A)** body weight and **(B)** organ index in mice.

As described in Figure 1A, the HFD group mice markedly raised the body weight compared to the Con group (*p* < 0.05). An 8-week treatment with HEO markedly decreased body weight compared to the HFD group (*p* < 0.05), but medium and low doses of AAEO did not affect body weight in ICR mice (*p* > 0.05). As described in Figure 1B, the low, medium, and high doses of AAEO did not markedly change SAT, AAT, EAT, PEAT, and BAT weight in ICR mice (*p* > 0.05). Although HFD markedly promoted liver weight compared to the control group (*p* < 0.05), the addition of high, medium, and low doses of AAEO did not dramatically affect liver weight in the HFD group (*p* > 0.05).

### Indicator of serum lipid metabolism

**FIGURE 2:**
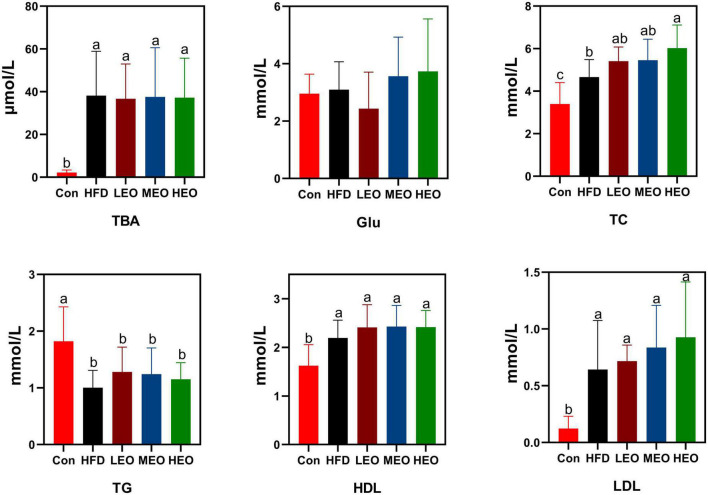
Effects of AAEO on serum lipid levels in mice.

As demonstrated in Figure 2, the HFD, LEO, MEO, and HEO raised serum TBA levels compared to the Con (*p* < 0.05). However, the addition of low, medium, and high doses of AAEO did not dramatically affect TBA level compared to the HFD group (*p* > 0.05). In addition, the HFD, LEO, MEO, and HEO declined in TG level compared to the Con (*p* < 0.05). However, the addition of low, medium, and high doses of AAEO did not dramatically affect TG levels compared to the HFD group (*p* > 0.05). There was no remarkable difference in Glu concentration in Con, HFD, and AAEO groups (*p* > 0.05). Although LEO- and MEO-fed ICR mice had no remarkable difference in TC level from the HFD group (*p* > 0.05), the HEO markedly enhanced the level of TC in blood compared with the HFD group (*p* < 0.05). All the AAEO treatments markedly raised the level of TC in blood compared with the Con group (*p* < 0.05). Meanwhile, the HFD, LEO, MEO, and HEO raised HDL levels compared to the Con (*p* < 0.05). However, the addition of low, medium, and high doses of AAEO showed no remarkable effect on HDL levels compared to the HFD group (*p* > 0.05). The HFD markedly raised LDL levels in ICR mice with or without AAEO supplementation (*p* < 0.05), and there was no remarkable effect on blood LDL levels between high, medium, and low doses of AAEO (*p* > 0.05).

### Histomorphological analysis of jejunum

**FIGURE 3:**
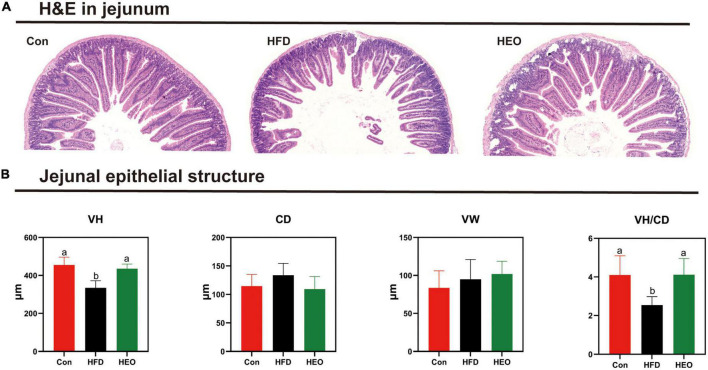
Effects of AAEO on the morphology of jejunal epithelial tissue in mice. **(A)** Light microscopy cross-section of the jejunal tissue. **(B)** The structure of the jejunal epithelial tissue.

Figure 3A shows the morphology of jejunal tissue under Con, HFD, and HEO treatments in ICR mice. In this study, jejunal villus height notably declined with the high-fat group compared to the Con diet-fed to ICR mice as described in Figure 3B (*p* < 0.05), but a high dose of AAEO induced a notably higher jejunal villus height than HFD in mice (*p* < 0.05). In ICR mice, the HFD fed to mice did not notably increase crypt depth in the jejunum compared to the Con group (*p* > 0.05), and there was no noteworthy change between the HEO, Con, and HFD groups (*p* > 0.05). With the high-fat diet fed to mice, the VH/CD declined in ICR mice compared to the Con diet (*p* < 0.05), and the VH/CD of the HFD group was notably lower than that of the AAEO group (*p* < 0.05). No matter the control group, high-fat group, or AAEO group, there was no noteworthy effect on the jejunal villus width of ICR mice (*p* > 0.05).

### Bacterial diversity in feces

**FIGURE 4:**
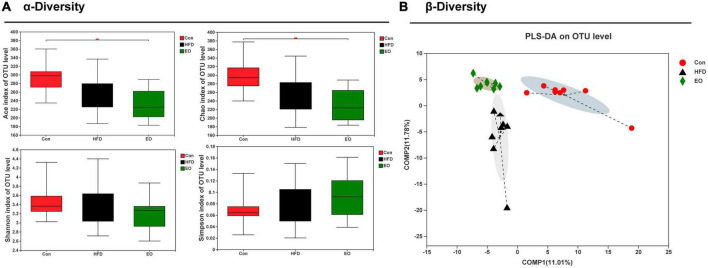
Effects of AAEO on **(A)** α diversity and **(B)** β diversity in the fecal microbiota of mice.

Based on three dietary treatments, Figure 4A shows the differences in fecal bacterial diversity among ICR mice. The study demonstrated that the EO fed to mice remarkably decreased the ACE index of OTU levels compared with the Con diet (*p* < 0.05). The three treatments did not affect fecal Shannon index of OTU levels in ICR mice (*p* > 0.05). Although the addition of EO in the diet had no marked effect on the HFD group on Chao index, while EO group remarkably decreased the Chao index compared with the Con group (*p* < 0.05). There was no notable change on the Simpson index of OTU level between the three different dietary treatments (*p* > 0.05). As demonstrated in Figure 4B, the male mice affected by the three dietary treatments produced significant segregation of PLS-DA on an OTU level.

### Fecal microbial composition

**FIGURE 5:**
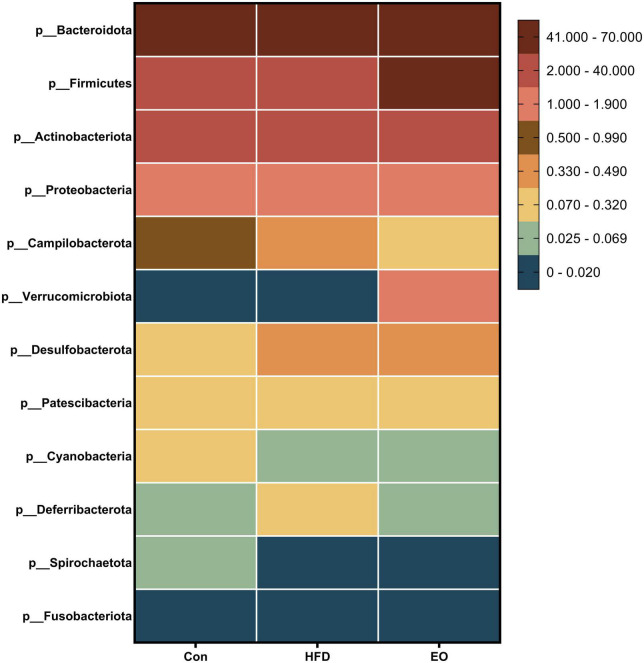
Effects of AAEO on phylum-level bacteria in the fecal microbiota of mice.

**FIGURE 6:**
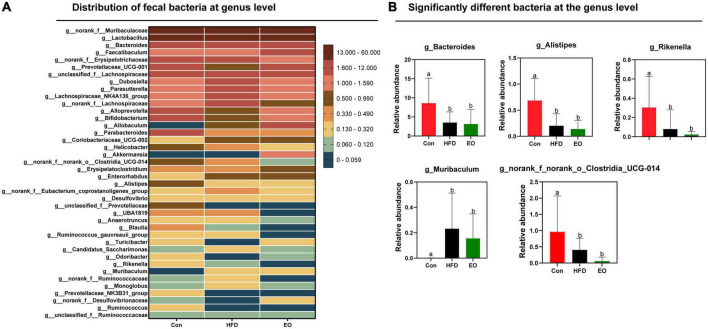
Effects of AAEO on **(A)** genus-level bacteria distribution and **(B)** significantly different bacteria at the genus level in the fecal microbiota of mice.

Figure 5 shows how the three treatments affected mice’s fecal microbiota at the phylum level (>1%). *Bacteroidota*, *Firmicutes*, *Actinobacteriota*, and *Proteobacteria* were the main phyla in the feces of mice, accounting for more than 98% of the total number of fecal bacteria. At the dominant phylum level, no differences were found in fecal bacteria from mice (*p* > 0.05). As described in Figure 6, *norank_f__Muribaculaceae*, *Lactobacillus*, *Bacteroides*, *Faecalibaculum*, and *norank_f__Erysipelotrichaceae* were major bacteria in the Con, HFD, and EO groups at the genus level. There were marked changes in 5 of the top 40 genera throughout the whole stage. The relative abundance of *Bacteroides* was greater (*p* < 0.05) in the feces from the Con diet when compared to the HFD and EO diets. On the contrary, the abundance of *Muribaculum* was lower in the Con diet than in the HFD and EO diets (*p* < 0.05). In the meantime, the relative abundance of *Alistipes* (*p* < 0.05) and *Rikenella* (*p* < 0.05) were also notably higher in the Con diet than in the HFD and EO diets. The abundance of *norank_f__norank_o__Clostridia_UCG-014* was lower in the HFD or EO diet than in the Con diet (*p* < 0.05). Although the abundance of *Muribaculum* in the EO diet was lower than that in the HFD diet, there was no notable difference (*p* > 0.05).

## Discussion

At present, the research in traditional Chinese medicine (TCM) focuses more on polysaccharides and flavonoids (31, 32). Researchers have demonstrated that a variety of dietary components can be used to treat obesity, including polysaccharides, polyphenols, terpenes, and alkaloids (33, 34). In addition, dietary amino acids and uridine can also affect body fat catabolism (35–37). The anti-obesity mechanisms usually included appetite suppression, fat absorption reduction, lipolysis enhancement, lipolysis reduction, and modification of the gut microbiota (38, 39). The leaf of *A. argyi* was widely used in TCM for its antimicrobial properties, relief from itching, and improvement of blood circulation. Compositae plants had a strong aromatic smell that attracted researchers to investigate their volatile composition (16). This study examined the effects of AAEO on fat deposition, blood lipid metabolism, fecal microbiota, and epithelial structure of jejunum in ICR mice. The HFD remarkably raised the final weight of the mice than their basal diet, while HEO restored the mice’s weight to the level of their basal diet. It showed that AAEO had a certain alleviating effect on obesity in mice. According to the results of fat deposition in this experiment, AAEO did not affect the fat weight of ICR mice, but AAEO did affect the weight of the liver, and the mechanism needed further experiments to clarify.

The blood biochemical indexes of the host not only provide information about the health and immune function of the body but also reveal their biological characteristics (40). There was a correlation between hypertrophymia and TC levels in the blood. Compared with LDL, HDL is more likely to cause hypertrophymia (41). In this experiment, AAEO did not change the concentrations of TBA, Glu, TG, HDL, and LDL in the blood of HFD mice; the high concentration of AAEO only raised the concentration of TC in the blood of HFD fed to mice. This indicated that AAEO had little effect on blood lipid metabolism in ICR mice.

Physical, chemical, microbiological, and immunological barriers make up the gut barrier (42). Dietary nutrients can modulate the small intestinal tissue morphology and digestive function of animals, and the gut barrier function is very critical to the host (43, 44). Nutrients were absorbed primarily through the small intestine. In evaluating small intestine digestion and absorption, villi height and crypt depth were key indicators. Deeper crypts reflected faster cell formation, whereas shallower crypts indicated accelerated maturation and improved secretion. It was possible to determine the functioning state of the gut by measuring the villus height and crypt depth (45). In recent years, physical barriers have been extensively examined in HFD resulting in increased intestinal permeability, which greatly increased the translocation of endotoxins from the gut to the blood circulation (46, 47). Throughout the intestine, tightly connected epithelial cells form a dynamic permeability barrier that prevents potentially harmful substances and allows nutrients into the blood circulation (42). In this study, HFD reduced villus length and increased crypt depth in mice, suggesting a disruption of the physical barrier of the jejunum. Meanwhile, HFD decreased the VH/CD in the jejunum of mice, but AAEO restored it, so the effect of AAEO on jejunal function may be positive.

The study of fecal microbiota is very necessary for the growth and health of animals (25, 30). Blood lipids are closely associated with gut microbiota, and normal gut microbiota may regulate blood lipid levels (48). Therefore, the main phylum-level flora of the mouse gut in this study was not affected by AAEO, corresponding to the same unaffected blood lipids in the experiment. To achieve nutrient absorption and deposition, the intestinal structures and microbiota must remain intact in order to induct chemicals and maintain digestive functions. The intestinal bacterial community was considered to have an important role in preserving intestinal function (49, 50). There were more than 100 trillion microorganisms in the ecosystem, most of which were bacteria (51). Microbiota composition and activity in the intestine were affected by many factors, including age, environment, and diet, with diet being the most crucial of these (52–54). Gut microbiota colonization and gut microbiota-mediated immunity are both influenced by diet (55). We explored the effect of AAEO on the bacteria community in feces. Based on the findings of the current study, HFD mice had lower bacterial diversity than ICR mice on a basal diet. Due to the high variability between and within species in the gut microbiome, it was difficult to define a healthy gut microorganism in terms of specifications (56, 57). Despite this, gut microbes and metabolites may be relatively stable (57, 58). According to previous studies, the ratio of Firmicutes to Bacteroides was increased in obese host (59) and correlated with host energy intake (60, 61). We found that 5 of the top 40 genera were notably altered over the course of the entire stage. The relative abundance of Bacteroides, Alistipes, Rikenella, and norank_f__norank_o__Clostridia_UCG-014 was lower in the HFD fed to ICR male mice in comparison with the control diet given to the mice. However, the addition of AAEO had no effect on these four bacteria. Simultaneously, the abundance of Muribaculum was higher after feeding HFD than after feeding the control diet, but adding AAEO on the basis of HFD had no effect on Muribaculum. This proved that AAEO had little effect on the main microbiota of mouse feces. So, we inferred that AAEO will not broadly regulate the intestinal microbial community.

## Conclusion

This study demonstrated that HFD could alter lipid metabolism and gut microbiota in ICR mice. Simultaneously, the gut barrier was weakened by HFD, which may impair the ability of nutrient absorption and digestion for the host. Although AAEO did not affect lipid deposition in mice, AAEO improved intestinal tissue morphology in mice. This study, which comprehensively investigated lipid metabolism, intestinal barrier, and the microbial response of AAEO in mice, indicated that AAEO was mainly beneficial to the intestinal barrier of mice.

## Data availability statement

The data presented in the study are deposited in the NCBI Sequence Read Archive (SRA) repository, accession number PRJNA861869.

## Ethics statement

The animal experiments were conducted in accordance with the Hunan Agricultural University Institutional Animal Care and Use Committee (202105).

## Author contributions

KW and ML designed the experiment. KW conducted the experiment and wrote the manuscript. KW, SX, JM, YL, QH, ZY, MZ, and JC collected and analyzed the data. SX revised the manuscript. All authors contributed to the article and approved the submitted version.

## Footnotes

1
https://dataview.ncbi.nlm.nih.gov/object/PRJNA861869
