# Corrigendum: Effects of essential oil extracted from *Artemisia argyi* leaf on lipid metabolism and gut microbiota in high-fat diet-fed mice

**DOI:** 10.3389/fnut.2024.1416210

**Published:** 2024-06-13

**Authors:** Kaijun Wang, Jie Ma, Yunxia Li, Qi Han, Zhangzheng Yin, Miao Zhou, Minyi Luo, Jiayi Chen, Siting Xia

**Affiliations:** ^1^College of Animal Science and Technology, State Key Laboratory for Conservation and Utilization of Subtropical Agro-Bioresources, Guangxi University, Nanning, Guangxi, China; ^2^Animal Nutritional Genome and Germplasm Innovation Research Center, College of Animal Science and Technology, Hunan Agricultural University, Changsha, Hunan, China; ^3^Agricultural Service Center, Zhongshan, Guangdong, China; ^4^Academician Workstation, Changsha Medical University, Changsha, Hunan, China

**Keywords:** *Artemisia argyi*, high fat, lipid, gut, microbiota

In the published article, there was an error in affiliation(s) [1] and [2]. Instead of “[1] Animal Nutritional Genome and Germplasm Innovation Research Center, College of Animal Science and Technology, Hunan Agricultural University, Changsha, Hunan, China. [2] College of Animal Science and Technology, State Key Laboratory for Conservation and Utilization of Subtropical Agro-bioresources, Guangxi University, Nanning, Guangxi, China”, it should be “[1] College of Animal Science and Technology, State Key Laboratory for Conservation and Utilization of Subtropical Agro-bioresources, Guangxi University, Nanning, Guangxi, China. [2] Animal Nutritional Genome and Germplasm Innovation Research Center, College of Animal Science and Technology, Hunan Agricultural University, Changsha, Hunan, China.”

The authors apologize for this error and state that this does not change the scientific conclusions of the article in any way. The original article has been updated.

